# In Vitro Development of a New Sponge-Based Delivery System for Intracanal Antimicrobial Administration in Endodontic Treatment

**DOI:** 10.3390/jcm10122725

**Published:** 2021-06-21

**Authors:** Ruíz-Piñón Manuel, Gancedo-Gancedo Tania, Seoane-Prado Rafael, Pérez-Estévez Antonio, Blanco-Méndez José, Luzardo-Álvarez Asteria, Castelo-Baz Pablo, Lorenzo-Pouso Alejandro, Álvarez-Novoa Pablo, Martín-Biedma Benjamín

**Affiliations:** 1 Faculty of Medicine and Odontology, University of Santiago de Compostela, 15705 Santiago de Compostela, Spain; manuelrz2@gmail.com (R.-P.M.); rafael.seoane@usc.es (S.-P.R.); antonio.perez.estevez@usc.es (P.-E.A.); pablocastelobaz@hotmail.com (C.-B.P.); alexlopo@hotmail.com (L.-P.A.); pablo.alvarez.novoa@gmail.com (Á.-N.P.); b.martinbiedma@gmail.com (M.-B.B.); 2 Faculty of Pharmacy, University of Santiago de Compostela, 15782 Santiago de Compostela, Spain; jose.blanco.mendez@usc.es; 3 Faculty of Sciences, University of Santiago de Compostela, 27002 Lugo, Spain; asteriam.luzardo@usc.es; 4 Paraquasil Group, University Clinical Hospital, Health Research Institute of Santiago de Compostela (IDIS), 15706 Santiago de Compostela, Spain

**Keywords:** sponge, delivery system, intracanal, antimicrobial, endodontic treatment

## Abstract

This study aimed to evaluate the in vitro performance of collagen-based sponges as a drug delivery system for intracanal antimicrobial administration. Four groups of loaded collagen-based sponges (A, 0.3% *w*/*v* amoxicillin trihydrate: potassium clavulanate (4:1); B, 0.03% *w*/*v* chlorhexidine gluconate [CHX]; C, 0.3% *w*/*v* amoxicillin trihydrate: potassium clavulanate (4:1) and 0.03% *w*/*v* CHX; D, 1% *w*/*v* amoxicillin trihydrate: potassium clavulanate (4:1) and 0.03% *w*/*v* CHX) were designed. Release kinetics were tested in vitro on cultures in Petri dishes, and the effect on bacterial biofilms was studied ex vivo on 114 extracted human single-rooted teeth. Biofilm formation was tested by scanning electron microscopy (SEM). Collagen sponges containing amoxicillin and chlorhexidine showed a time-sustained antimicrobial effect in vitro and were also able to destroy mature biofilms ex vivo. This datum was validated by means of SEM-based study of *E. faecalis* and *S. aureus* biofilms.

## 1. Introduction

Root canal instrumentation, irrigation, and frequently co-adjuvant intracanal medication are relevant prerequisites in non-surgical endodontic treatment. These methods are clinically used to reduce the number of bacteria within the root canal system [1,2,3,4]. In this line, several complications that can jeopardize non-surgical endodontic treatment related success have been described, such as the presence of inaccessible anatomical irregularities and the endodontic pathogens’ ability to form biofilms [1,2,5,6,7]. These two characteristics represent limiting factors in the fight against these pathogens. This reality transferred into clinical practice plays a pivotal role in the origin of endodontic failure [1]. 

Intracanal medication, as reported previously, had a high success rate in teeth diagnosed with pulpal necrosis and apical periodontitis. Single-appointment endodontic treatment has a lot of advantages; however, in cases with apical pathosis or long-term radiographic lesions, it is not possible to ensure that healing will begin once endodontic therapy is completed. Therefore, in these cases, disinfection should be enhanced [8]. 

Antiseptics and antibiotics are therefore frequently used as root canal dressing between appointments [3,6]. Antiseptics are usually introduced into the canal, but antibiotics are administered systemically and do not reach concentrations high enough to prevent the growth of resistant bacteria such as *E. faecalis*. The local application of the antibiotics is more effective than a systemic one [3,9]. The relationship between *E. faecalis* and endodontic failure is well-documented [2,9]. The ability of *E. faecalis* to form biofilms increases its resistance to commonly administered antimicrobial treatments, and various approaches have therefore been used to enhance the anti-enterococcal effect of these treatments within the root canal [9]. Several modifications to irrigation regimens, including the addition of antibiotics, antiseptics, and/or surfactants—such as, for example, CHX-Plus (Vista Dental Products, Racine, WI), which is a combination of chlorhexidine digluconate with surface modifiers; MTAD (BioPure MTAD, Dentsply Sirona Endodontics, York, PA, USA), which is a mixture of an antibiotic (doxycycline), citric acid and a detergent; or Qmix (Dentsply Tulsa Dental Specialties, Tulsa, OK), which is a combination of CHX, EDTA and a detergent—all show variable degrees of activity against this bacterium [2,10]. The design of new vehicles and antimicrobial combinations for intracanal medication effective against *E. faecalis* is thus an expanding and exciting field [2,6]. In the past, our group developed biocompatible amoxicillin-collagen sponges and showed that cross-linking improved water uptake, mechanical properties, and cell compatibility when compared to non-crosslinked specimens [11] and polymeric biodegradable microspheres of D-L lactide-co-glycolide able to deliver amoxicillin in a sustainable manner for root canal disinfection were developed [12]. The sponge delivery systems exhibit an optimal biocompatibility, easy management that does not require posterior removal and antimicrobial superiority against *E. faecalis* over calcium hydroxide (the substance most commonly used as a canal dressing between appointments) [13,14]. 

In this study, the in vitro antimicrobial release kinetics of collagen sponges impregnated with different antimicrobial formulations and their activity against mature biofilms in agar plates and in extracted teeth were evaluated. The aim is to demonstrate the effectiveness of the association between chlorhexidine and amoxicillin and corroborate that with a lower concentration of antibiotics, similar results could be obtained. The null hypothesis was that there are no differences in the antimicrobial effect exerted between the different formulations used and that the effect of the chlorhexidine and amoxicillin was synergistic.

## 2. Materials and Methods

### 2.1. Ethics Approval

All procedures performed in this study were in accordance with the ethics standards of the institutional and national research committee and with the 1964 Helsinki declaration. The work was approved by Ethical Committee of University of Santiago de Compostela, No. 2016/269.

### 2.2. Preparation of Collagen Sponges

Each sponge was assembled as previously described by our group [11]. In summary, calf-skin type-1 collagen (CL) (Fagrón Ibérica SAU, Barcelona, Spain), poly[(methyl vinyl ether)-co-(maleic anhydride)] (Gantrez^®^ MS-955 (PVMMA)) and Tween^®^ 80 (Sigma-Aldrich, St. Louis, MO, USA) as surfactant were dissolved in water at 0.38% and 0.0038%, respectively [11]. Control sponges were obtained directly from this solution without any additives. Test sponges were obtained by the inclusion of the following antimicrobial agents: (A) 0.3% *w*/*v* amoxicillin trihydrate: potassium clavulanate (4:1) (Sigma-Aldrich, St. Louis, MO, USA); (B) 0.03% *w*/*v* CHX (Sigma-Aldrich, St. Louis, MO, USA); (C) 0.3% *w*/*v* amoxicillin trihydrate: potassium clavulanate (4:1) (Sigma-Aldrich, St. Louis, MO, USA) and 0.03% *w*/*v* CHX (Sigma-Aldrich, St. Louis, MO, USA); (D) 1% *w*/*v* amoxicillin trihydrate: potassium clavulanate (4:1) (Sigma-Aldrich, St. Louis, MO, USA) and 0.03% *w*/*v* CHX (Sigma-Aldrich, St. Louis, MO, USA). 

To establish the correct pH for the preparation of collagen sponges, the isoelectric point of collagen was evaluated, measuring the absorbance of 10 collagen dispersions prepared in 0.1 M acetate buffer, 0.1 M acetic acid or 0.01 M acetic acid. After an exhaustive analysis of the results, the sponges were finally prepared at pH 3.5, under which conditions PVMMA is negatively charged. To evaluate the appropriate CL/PVMMA proportion, a 2.5 mg/mL solution of CL in 0.5 M acetic acid and a 20 mg/mL solution of PVMMA in the same solvent were mixed at 25 °C to obtain mixtures with a CL concentration of 0.1% (*w*/*w*) and PVMMA concentrations ranging from 0.01 to 0.5% (*w*/*w*). The optimal CL/PVMMA ratio was determined on the basis of turbidometric and conductimetric measurements of these mixtures.

Formulations were prepared with a CL/PVMMA ratio of 1:0.75 (*w*/*w*) and the different antimicrobial solutions were added. These different solutions were then frozen at −80 °C in 330 μL aliquots and Cryodos (Telstar) lyophilized to obtain ready-to-use sponges. All experiments were performed within four weeks of freezing. After preparation of the sponges, the study was performed in two phases:

### 2.3. First Phase: In Vitro Assay into Agar Plates

*E. faecalis* ATCC 29212 and *S. aureus* ATCC 292135 strains were purchased from the Spanish Collection of Type Cultures (CECT), cultured anaerobically overnight at 37 °C in brain heart broth (BHI) (Difco, Becton Dickinson, Sparks, MD, USA), adjusted to 0.5 McFarland units, and used to inoculate Mueller-Hinton agar (MHA) plates. Immediately after inoculation, sterile 6-mm-diameter paper disks were positioned on the agar, and sponges were carefully placed on these disks. After 24 h of anaerobic incubation at 37 °C, inhibition halos were measured, and the sponge-supporting disks were transferred to freshly inoculated plates.

Equivalent amounts of amoxicillin were determined by means of a standard regression obtained from inhibition by disks containing 150, 100, 75, 50, 25, 12.5, 6.25, 3.125, 1.56, and 0.78 μg of pure amoxicillin (Sigma-Aldrich, St. Louis, MO, USA). 

### 2.4. Second Phase: Preparation of Tooth Specimens

A total of 120 single-rooted human teeth (maxillary central incisors with one oval-shaped root canal and substantially equal canal curvature), with fully formed apices that had not undergone prior endodontic treatment, extracted due to periodontal reasons were used. The protocol used during this ex vivo phase for tooth preparation was described previously by our group [7]. In brief, after root surface debridement, specimens were immersed in a 5.25% NaOCl solution (Niclor 5; OGNA, Muggiò, Italy) for 1 h and then stored in saline solution for 1 day at 37 °C until preparation. The presence of a single canal was verified radiographically and by direct exploration. The same operator performed all experimental procedures. Each specimen was sectioned to obtain a working length of 16 mm. The working length (taken 1 mm short from the apical foramen) was established under a microscope (M525 F40; Leica, Heerbrugg, Switzerland) at 10× magnification with the tip of the instrument, a #10 K-Flexofile (Dentsply Maillefer, Ballaigues, Switzerland), visible at the apical foramen. The patency of each root canal was assessed using K-Flexofiles (Dentsply Maillefer, Ballaigues, Switzerland) up to #20. In this study, all the canals were prepared using ProTaper S1-S2-F1-F2-F3-F4 (PTG; Dentsply Maillefer, Ballaigues, Switzerland) [7].

Irrigation was performed with a 30-gauge needle (ProRinse; Dentsply Tulsa Dental Specialties, Tulsa, OK, USA) using 3 mL of 5.25% NaOCl after each file. The irrigation needles were introduced passively up to 2 mm from the working length, and the rate of delivery was fixed at 3 mL/min. The total irrigation time was 10 min/specimen. After instrumentation, all teeth were rinsed for 3 min with 3 mL of 10% EDTA (Tubuliclean; OGNA, Milan, Italy) followed by a 3-min final rinse with 5.25% NaOCl, activating both solutions with an ultrasonic tip for 30 s (Irri-Safe; Satelec, Acteón Group, Merignac Cedex, France). A final wash was made with distilled water. After drying with paper points, the roots were inspected under the microscope at 10× magnification to verify the absence of cracks and canal cleanliness [7]. 

After the completion of the shaping procedures, the apical foramen was sealed using light-cured restorative composite (CeramX Duo, Dentsply-Friadent, Roskilde, Denmark). Each specimen was transferred to a plastic cryo-tube (Cryo. S, PP Greiner Bio-One Gmbh., Frickenhausen, Germany) containing sterile brain heart infusion (BHI) broth (Merck KGaA, Darmstadt, Germany) and autoclaved at 121 °C for 20 min.

#### 2.4.1. Experimental Root Canal Infection

An overnight pure culture of *E. faecalis* and *S. aureus* in BHI (Merck KGaA, Darmstadt, Germany) was used for inoculation. The bacterial suspension was adjusted spectrophotometrically to match the turbidity of a McFarland 0.5 scale. Aliquots of 20 μL of the suspension were inoculated into each canal, then specimens were coronary sealed with composite (CeramX Duo, Dentsply-Friadent, Roskilde, Denmark) and incubated for 21 days under aerobic conditions at 37 °C into Falcon tubes (Eurolab, Barcelona, Spain) with 5 mL of BHI, changing it every three days. Two teeth inoculated with sterile BHI (Merck KGaA, Darmstadt, Germany) were used as negative controls (*n* = 2). 

After 21 days, the tooth surfaces were disinfected with CHX swabs and then washed profusely with saline solution to neutralize CHX. After removal of the composite and cotton sealing, two samples were taken with sterile paper tips and incubated in BHI broth (Merck KGaA, Darmstadt, Germany) at 37 °C to confirm infection (positive controls, *n* = 2). 

Two sterile tips were introduced in the specimens during 30 s and then incubated in 5 mL of BHI at 37 °C. After 24 h, infection was confirmed by turbidimetry (Dinko D-101, Barcelona, Spain; at 590 ηm of wavelength) and CFU count. Briefly, after tenfold serial dilution in saline, aliquots of 0.1 mL were plated into BHI agar plates and incubated at 37 °C for 48 h. The colony-forming units (CFUs) grown were counted and then transformed into actual counts based on the known dilution factors. All samples showed bacterial growth at 48 h. 

**Figure 1:**
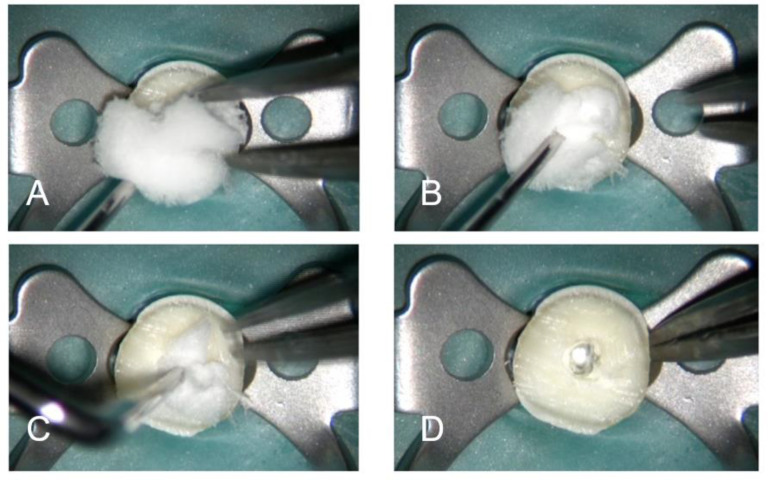
(**A**) Placement of the sponge at the canal entry. (**B**,**C**) Compaction at the coronal and middle third of the root. (**D**) Collagen sponge properly placed at the apical third of the root.

**Figure 2:**
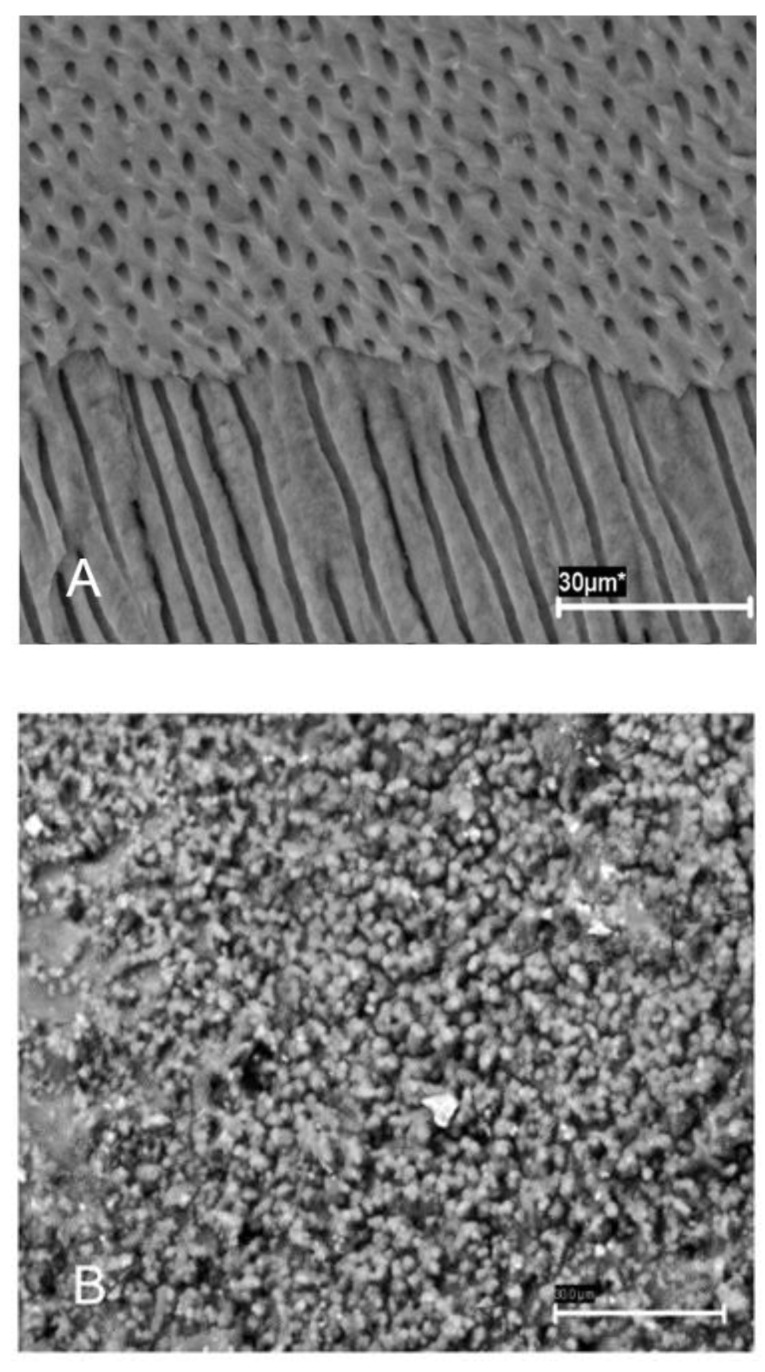

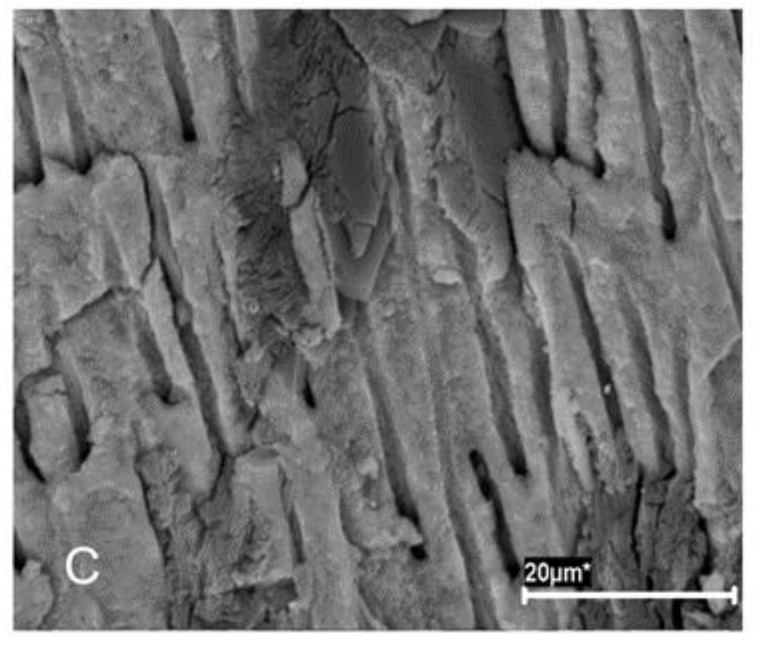
Scanning electron micrograph of dentinal surfaces. (**A**) Uninfected teeth. (**B**) Mature biofilms 21 days post-infection. (**C**) Clean dentine after sponge removal in infected teeth.

Experimental sponges (Figure 1) were then introduced with a vertical condensation plugger, and the teeth were resealed as above and incubated in BHI broth (Merck KGaA, Darmstadt, Germany) at 37 °C. The formation of biofilms was confirmed by scanning electron microscopy (SEM) (Figure 2A,B) and colony-forming unit (CFU) count. 

#### 2.4.2. Experimental Groups and Treatment (Agar Plates and Tooth Specimens)

Group 1 (*n* = 14): A (0.3% *w*/*v* amoxicillin trihydrate: potassium clavulanate (4:1)) against *E. faecalis.*

Group 2 (*n* = 14): B (0.03% *w*/*v* chlorhexidine gluconate [CHX]) against *E. faecalis*.

Group 3 (*n* = 14): C (0.3% *w*/*v* amoxicillin trihydrate: potassium clavulanate (4:1) and 0.03% *w*/*v* CHX) against *E. faecalis*.

Group 4 (*n* = 14): D (1% *w*/*v* amoxicillin trihydrate: potassium clavulanate (4:1) and 0.03% *w*/*v* CHX) against *E. faecalis*.

Group 5 (*n* = 14): A (0.3% *w*/*v* amoxicillin trihydrate: potassium clavulanate (4:1)) against *S. aureus*.

Group 6 (*n* = 14): B (0.03% *w*/*v* chlorhexidine gluconate [CHX]) against *S. aureus*.

Group 7 (*n* = 14): C (0.3% *w*/*v* amoxicillin trihydrate: potassium clavulanate (4:1) and 0.03% *w*/*v* CHX) against *S. aureus*.

Group 8 (*n* = 14): D (1% *w*/*v* amoxicillin trihydrate: potassium clavulanate (4:1) and 0.03% *w*/*v* CHX) against *S. aureus*.

### 2.5. Scanning Electron Microscopy (for Tooth Specimens)

Four root specimens were selected for SEM to confirm the established biofilm infection. Each specimen was split into two halves, with a pointed stainless-steel chisel inserted in pre-fracture grooves, and fixed in 5 mL of 3.7% glutaraldehyde in 0.2 M sodium cacodylate buffered solution at 4 °C for 24 h. Following dehydration in graded concentrations of ethanol, specimens were air dried and mounted on SEM stubs for gold sputtering and observation with a JEOLJSM 6400 scanning electron microscope (JEOL Corporation, Tokyo, Japan). SEM microphotographs were obtained at different magnifications in representative areas of the root canal system.

### 2.6. Determination of the Number of Bacteria (for Tooth Specimens)

The specimens were processed at two intervals of time, 24 h and 12 days after the experimental disinfection procedures. After opening the teeth, the canals were refilled with normal saline as a transfer fluid. Sampling from inside the canals consisted of using a sterile #25 K-File (Dentsply, Maillefer) with circumferential filing for 20 s in order to disrupt the biofilm and to collect dentin chips. Sterile paper points were used to collect the transfer fluid and dentin chips. Both sterile paper points and sampling K-files were placed into a test tube containing 4 mL of sterile BHI and vortexed for 1 min.

After sampling, 20 μL of sterile saline solution was introduced in samples, and then they were coronally sealed with composite. Specimens were then sealed again with composite and incubated in BHI up to 12 days. The previously described procedure was performed once more at 12 days. The CFUs were calculated. The colonies were confirmed to be *E. faecalis* and *S. aureus* by colony morphology, Gram staining, and PCR.

### 2.7. Statistical Analysis

The variables showed no normal distribution according to the Kolmogorov–Smirnov test. Quantitative data are expressed as medians and range. Comparisons of CFU values between groups were analyzed by Kruskal–Wallis and Chi-square tests with Bonferroni correction. *p* values < 0.05 were considered statistically significant. Analyses were performed using the SPSS program (SPSS INC, Chicago, IL, USA) version 17.0.

## 3. Results

### 3.1. First Phase: In Vitro Assay into Agar Plates

**Figure 3:**
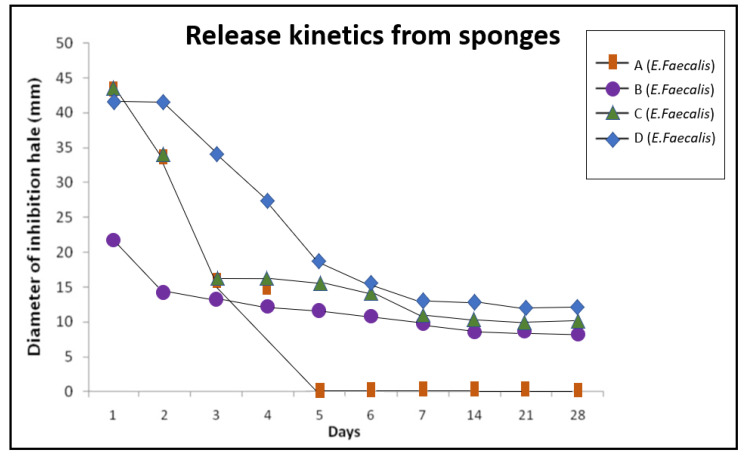
Release kinetics from sponges. Inhibition halos obtained on days against *E. faecalis*; square: sponge A; circle: sponge B; triangle: sponge C; rhombus: sponge D.

**Figure 4:**
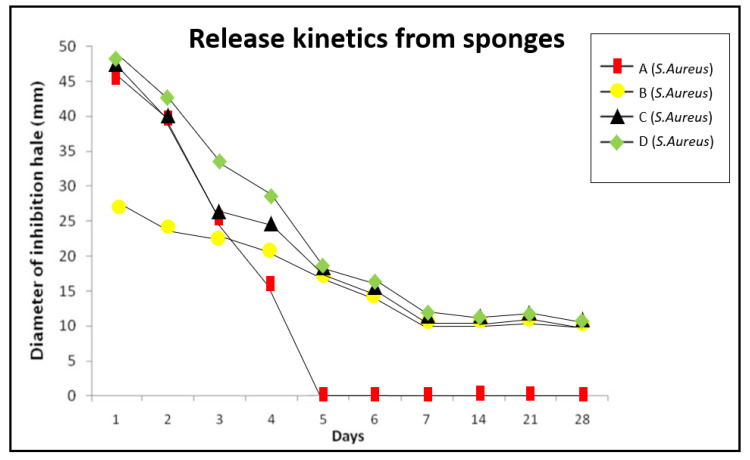
Release kinetics from sponges. Inhibition halos obtained on days against *S. aureus*; square: sponge A; circle: sponge B; triangle: sponge C; rhombus: sponge D.

Figure 3 and Figure 4 show the time course of inhibition halos induced by the different sponges against *E. faecalis* (Figure 3) and *S. aureus* (Figure 4). Amoxicillin, in sponges A and C, had a highly antimicrobial effect during the first four days; amoxicillin release from the D sponges were sustained for five days, but after day five, the release pattern was remarkably similar to that of the C sponges. In contrast, CHX released from sponges B, C and D was more uniform and persisted for more than 28 days. No increase in activity was detected with CHX/amoxicillin sponges C and D, indicating that the effects of amoxicillin and CHX were complementary, not synergistic, and that with a lower concentration of antibiotic, satisfactory results could be obtained. 

### 3.2. Second Phase: Preparation of Tooth Specimens

The absence of growth was seen in the negative controls (two teeth inoculated with sterile BHI). All specimens of the eight groups showed bacterial growth prior to the experimental disinfection procedures as confirmed by colony-forming unit (CFU) count and scanning electron microscopy (SEM) with four root specimens. The median of CFUs is shown in Table 1, and successively, there were no statistically significant differences between the eight groups. 

In teeth treated with sponges A and B, two of 14 teeth (14.3%) showed bacterial growth within the first 12 days after treatment. In contrast, no growth was detected in the teeth treated with either of the CHX/amoxicillin-clavulanate sponges (C and D). Furthermore, SEM showed the complete disappearance of the enterococcal biofilms in the teeth treated with the C and D sponges (Figure 2C), demonstrating its efficiency. The four teeth treated with control sponges showed positive growth on day 12.

## 4. Discussion

The null hypothesis was rejected due to the fact that the results showed that the inhibition halos and antimicrobial effects varies between the different formulations used and the CHX and amoxicillin demonstrate a synergistic character. 

*E. faecalis* and *S. aureus* are Gram-positive cocci, facultative anaerobes that have a great potential to form biofilms on different substrates; this ability has been associated with their persistence after primary endodontic treatment on numerous occasions [4,9,15,16]. The organization of bacteria in biofilms, and dynamic and complex environments provides increased resistance against the elements intended for their eradication, compromising their effectiveness [2,15,16].

Intracanal medication is a disinfection tool in addition to the instrumentation and irrigation of the root canal system, reaching areas inaccessible to them and acting for a longer period of time [17]. Calcium hydroxide continues to be the most widely known and used substance as intracanal medication in endodontics when is indicated [15,18]. However, despite its recognized advantages, it is not effective in the fight against *E. faecalis* and other persistent bacterial species [2,14,15,17,19]. The use of antibiotics for this purpose has also been described, especially the triantibiotic paste based on metronidazole, minocycline, and ciprofloxacin, widely used in regenerative endodontics; however, negative effects have been reported with their use as crown discoloration and the difficulty with its complete removal [19,20,21].

In a study conducted by Barbosa et al. [9] the antimicrobial efficacy of various antibiotics against *E. faecalis* was evaluated. Amoxicillin-clavulanate was effective in 100% of cases, followed by amoxicillin and penicillin G. Aminoglycosides were not clinically effective on *E. faecalis*, nor was clindamycin, azithromycin, erythromycin, or metronidazole. In contrast, good results were reported with chloramphenicol and moxifloxacin, so they could be options to evaluate in resistant cases or in patients allergic to penicillin and its derivates [9].

Sodium hypochlorite (NaOCl) is widely known as the most potent disinfectant in endodontics due to its excellent ability to dissolve organic tissue, in addition to its antimicrobial activity. However, as reported in a study by Valverde et al. [22] NaOCl is inactivated after a short time, so it is not expected to prevent bacterial regrowth; for this reason, its use as an intracanal medication is limited [22]. CHX, on the other hand, shows good behavior as an irrigant or as an intracanal medication, due to its biocompatibility and antimicrobial activity [18]. Xu et al. [4], observed that when CHX was used as the final irrigant solution for 1 min, the adherence of *E. faecalis* to the dentinal surface was lower [4]. The most remarkable property of CHX is its substantivity; in addition, it is efficient in fighting microorganisms resistant to calcium hydroxide [4,18]. 

For all these reasons, biofilms of *E. faecalis* and *S. aureus* were used in this article to test the efficacy of CHX, amoxicillin, and amoxicillin with clavulanic acid; this represent an advantage over other studies that evaluate antimicrobial efficacy on planktonic bacteria, which are more susceptible than bacteria organized in biofilms. However, the in vitro character of the study represents an important limitation, since oral biofilms are polymicrobial in nature, while the ones used in this study are not; nevertheless, this study model is easily replicable and allows various modifications, providing the basis for studying their in vivo behavior [2].

Scanning electron microscopy (SEM) was used as the main method to confirm the correct state of the samples, as previously performed in other studies [4,19]. There are other techniques to analyze the structure of biofilms, such as confocal scanning electron microscopy or super-resolution fluorescence microscopy techniques; however, these techniques use fluorescent markers, which is controversial as the processing of the samples itself may cause cell death. SEM solves some of the limitations offered by fluorescent dyes, but sample preparation can produce some distortion in the extracellular polymer matrix, with environmental scanning electron microscopy (ESEM) being a more useful method [2].

In this study, the effects of collagen sponges were analyzed by combining the intense and rapid effect of the antibiotic with the substantivity of CHX. As can be observed in Figure 3 and Figure 4, the inhibitory effects of sponges A, B, C and D are similar during the first three days, suggesting that amoxicillin and CHX do not act synergistically but rather complement each other. The C and D sponges eliminated biofilms in 100% of the examined samples, showing to be a remarkably effective option to eliminate *E. faecalis* and *S. aureus* biofilms on the dentin surface. Group C exhibited a lower activity during the first four days, but then the inhibition halos obtained were similar of the group D. These results suggested that with a lower concentration of antibiotic, similar effectiveness could be obtained, and the apparition of resistances could be reduced, reserving the use of D sponges for refractory cases, for example. 

This study supports the use of collagen sponges as useful vehicles for the release of various substances. The sponges are dissolvable with the course of time (around seven days), so they do not require removal as with calcium hydroxide or antibiotic pastes. The removal of these substances from the canals is difficult to achieve, as reported in some previous studies [23,24]. Genipin-crosslinked CL-PVMMA sponges prepared as in this study have enough cytocompatibility, mechanical strength, plasticity, and ability to absorb fluids without disintegrating to support sustained drug delivery and cell growth for a sufficient period of time [11]. In addition to their antimicrobial efficacy, sponges are also being investigated in regenerative endodontics due to their cytocompatibility and ability to stimulate nucleation sites [25]. Nowadays, several mechanisms for sustained drug release are being investigated. One of these methods is the release of antibiotics by biodegradable polymer nanofibers that are very respectful of stem cells, which is especially useful in regenerative endodontic therapies [19]. On the other hand, nanomaterials enable to obtain the maximum therapeutic efficacy with the fewest possible adverse effects, due to their high reactivity [26]. In the field of endodontics, nanotechnology has especially focused on improving antimicrobial efficacy. Silver nanoparticles have been shown to be highly effective against *E. faecalis* [2,26]. Their use in combination with mesoporous materials that act as reservoirs represents a promising option [27].

In a study by Chen et al. [15] the utility of a bromophenol compound thihydantoin (ST056083) in the synthesis of c-di-AMP, a secondary messenger used in signal transduction between bacteria, was investigated. The inhibitory effect of ST056083 against DisA, a c-di-AMP synthetase, was observed, resulting in an impaired biofilm formation [15]. This method provides an alternative in the elimination of bacteria through the inhibition of biofilm growth since the effect of the existing antimicrobial substances is significantly higher on the bacteria in the planktonic form.

Additional in vivo studies are required to corroborate the results obtained in vitro, and a critical and constant review of the new bacterial resistances, since despite the high success rate obtained with primary endodontic treatment, around 90%, there is a computation of failures that may be due to various causes, especially microbiological ones [5,28].

## Figures and Tables

**Table 1:** Median and range specimens CFU prior to the treatment and number of infected teeth at 24 h and at day 12 after treatment.

Groups	CFU/mL: Median (×10^7^) and Range	Number of Infected Teeth at 24 h	Number of Infected Teeth at 12 Days
Group 1 (*n* = 14)	128 (51–312)	1	2
Group 2 (*n* = 14)	149 (77–327)	2	2
Group 3 (*n* = 14)	124 (80–182)	0	0
Group 4 (*n* = 14)	136 (62–280)	0	0
Group 5 (*n* = 14)	138 (95–207)	2	2
Group 6 (*n* = 14)	120 (87–154)	1	2
Group 7 (*n* = 14)	114 (44–302)	0	0
Group 8 (*n* = 14)	137 (90–181)	0	0
